# Neural Mechanisms Underlying the Effects of Novel Sounds on Task Performance in Children With and Without ADHD

**DOI:** 10.3389/fnhum.2022.878994

**Published:** 2022-06-21

**Authors:** Jana Tegelbeckers, André Brechmann, Carolin Breitling-Ziegler, Bjoern Bonath, Hans-Henning Flechtner, Kerstin Krauel

**Affiliations:** ^1^Department of Children and Adolescent Psychiatry and Psychotherapy, Otto-von-Guericke-University Magdeburg, Magdeburg, Germany; ^2^Department of Neuropsychology, Otto-von-Guericke-University Magdeburg, Magdeburg, Germany; ^3^Combinatorial Neuroimaging Core Facility, Leibniz Institute for Neurobiology, Magdeburg, Germany; ^4^Center for Behavioral Brain Sciences, Magdeburg, Germany

**Keywords:** ADHD (attention deficit hyperactivity disorder), alertness, distraction, executive control, fMRI, novelty, orienting

## Abstract

Distractibility is one of the key features of attention deficit hyperactivity disorder (ADHD) and has been associated with alterations in the neural orienting and alerting networks. Task-irrelevant stimuli are thus expected to have detrimental effects on the performance of patients with ADHD. However, task-irrelevant presentation of *novel* sounds seems to have the opposite effect and improve subsequent attentional performance particularly in patients with ADHD. Here, we aimed to understand the neural modulations of the attention networks underlying these improvements. Fifty boys (25 with ADHD) participated in a functional magnetic resonance imaging (fMRI) study in which unique (novel) or repeatedly presented (familiar) sounds were placed before a visual flanker task in 2/3 of the trials. We found that presenting any sound improved task performance in all participants, but the underlying neural mechanisms differed for the type of sound. Familiar sounds led to a stronger increase in activity in the left posterior insula in patients with ADHD compared to typically developing peers. Novel sounds led to activations of the fronto-temporoparietal ventral attention network, likewise in ADHD and TD. These changes in signaling by novelty in the right inferior frontal gyrus were directly related to improved response speed showing that neural orienting network activity following novel sounds facilitated subsequent attentional performance. This mechanism of behavioral enhancement by short distractions could potentially be useful for cognitive trainings or homework situations.

## Introduction

Attention-deficit/hyperactivity disorder (ADHD) is one of the most prevalent disorders in childhood and adolescence (Polanczyk et al., 2007). The clinical picture is characterized by age-inappropriate levels of impulsivity, motor activity, and deficient attentional capacity. The latter deficit manifests in severe difficulties to direct and maintain attention as well as a significantly enhanced distractibility.

Distractibility is however not exclusively associated with negative consequences but is an important mechanism to promote adaptive behavior. Thus, the potential positive impact of unexpected stimuli has been attributed to the contextual novelty of these distractors (e.g., SanMiguel et al., 2010; Wetzel et al., 2012; Schomaker and Meeter, 2014, 2015). Novel stimuli are highly salient as their appearance might indicate the necessity of behavioral adaptation to avoid harm or gain a potential reward. Therefore, novel as well as unexpected (contextually novel) stimuli elicit an automatic orienting response (Sokolov, 1963) and simultaneously increase alertness (Aston-Jones and Cohen, 2005). If the *alerting benefits* of the disruption exceed the *orienting costs*, behavioral facilitation can occur, otherwise performance detriments should be observable (SanMiguel et al., 2010).

This conceptual framework could be of significance for understanding different dysfunctions in attentional processes in ADHD. On the one hand, deficient inhibition of external stimulation has been thought to be responsible for the breakthrough of task-irrelevant information (Barkley, 1997), suggesting enhanced orienting costs (distractibility) in ADHD patients. On the other hand, openness to external stimulation has been viewed as a mean to overcome underlying deficits of arousal and alertness in ADHD (Zentall and Zentall, 1983; Sergeant, 2005). In this regard, alerting benefits, e.g., utilization of warning cues, can be of utmost importance.

Previous studies in ADHD patients revealed the divergent effects of task-unrelated, especially novel stimuli on behavior. Some studies reported the anticipated distracting effect of task-irrelevant stimuli on children, adolescents as well as adults with ADHD (e.g., Gumenyuk et al., 2005; Berger and Cassuto, 2014; Pelletier et al., 2016). Others showed, however, that a novel environment reduced hyperactive behavior in waiting situations (Antrop et al., 2000) and that novel sounds facilitated attentional processing in visual decision tasks in ADHD patients (van Mourik et al., 2007; Tegelbeckers et al., 2016). In these latter studies, novel sounds preceded the onset of a task-display so that the alerting effect of novelty could unfold optimally and reduce omission errors (van Mourik et al., 2007) as well as commission errors and reaction time variability (Tegelbeckers et al., 2016).

Most importantly, the beneficial effects of novel sounds seem to exceed the effect of a repeatedly presented, familiar sound (standard) on behavioral attentional performance (van Mourik et al., 2007; Tegelbeckers et al., 2016). Facilitation in this case can thus not purely be attributed to the preparatory signal provided by any sound cue. Instead, the unexpectedness of the unique sound seems to play a crucial role causing either enhanced *alerting* benefits or the *orienting* reaction, which is only triggered by novel but not familiar stimuli.

Here, we aimed for the first time to gain insight into the neural processes underlying the beneficial effects of task-preceding novel sounds in children and adolescents with but also without ADHD. Following SanMiguel et al. (2010), we expected that novel sounds operate *via* different attention related processes based on separate and distinguishable neural networks (Petersen and Posner, 2012).

The fronto-parietal *alerting network* is closely related to noradrenergic brain circuits and refers to the ability to maintain an alert state for a certain period of time (tonic) as well as to utilize external cues to increase the readiness to respond (phasic). The behavioral advantage of using phasic alerting cues seems to be unimpaired in children with ADHD as they benefit from task announcing information similar to typically developing (TD) children (e.g., Gupta and Kar, 2009; Casagrande et al., 2012; Fassbender et al., 2015). The level of tonic alertness is, on the other hand, supposedly decreased in ADHD (Zentall and Zentall, 1983; Sergeant, 2000) resulting in diminished sustained attention over time. Thus, the positive effect of task-preceding sounds in ADHD patients could be due to phasic activation increases of the tonic network.

Furthermore, two orienting networks are responsible for the voluntary direction of attention toward a task at hand (top-down) as well as the automatic allocation of attention toward changes in the environment (bottom-up) (Corbetta et al., 2008). These two functionally distinguishable components are subserved by different brain networks: bottom-up processing and automatic allocation of attention to salient sensory stimuli involves the temporo-parietal junction (TPJ) and ventral frontal cortex (ventral attention network, VAN) whereas top-down control of attention requires a dorsally located network (dorsal attention network, DAN) consisting of the intraparietal sulcus (IPS) and frontal eye field (FEF) (Corbetta and Shulman, 2002). Previous studies in ADHD revealed impairments during target processing which indicates deficits in controlled top-down orienting of attention (Kemner et al., 1996; Jonkman et al., 2000). The evidence for an ADHD related deficit in automatic orienting, on the contrary, remains scarce and inconsistent (Barry et al., 2003; Huang-Pollock and Nigg, 2003; Cortese et al., 2012). We previously found evidence for both, missing suppression of VAN activity as well as diminished habituation of novelty processing in ADHD patients when we disentangled effects of task relevance, rareness and novelty (Tegelbeckers et al., 2015). These results point to an elevated effect of novelty which might as well be associated with the beneficial behavioral effects of novel sounds in ADHD patients.

Here, we were interested in how alerting and/or orienting responses caused by novel sounds would influence brain activity and behavior during the subsequent execution of a visual flanker task (Tegelbeckers et al., 2016) in a group of typically developing children and adolescents as well as in a group of patients with ADHD who suffer from increased distractibility. To disentangle novelty related effects on task performance from mere alertness by a warning cue and to investigate the role of the proposed alerting deficit in ADHD (Sergeant, 2005), we compared the general alerting effect of a sound cue (*standard vs. no sound*) as well as the influence of *novel* sounds to the baseline without sound and extracted the specific novelty effect (*novel vs. standard sound*).

We expected that all participants benefit from sound presentations but that attentional performance is increased to a larger extent in ADHD patients for both warning sounds due to the alerting deficit associated with the disorder (Sergeant, 2005) and their enhanced susceptibility to novelty (Tegelbeckers et al., 2015, 2016). Thus, we also expected increased activity in the fronto-parietal alerting network in the patient group. Moreover, we hypothesized that novel sounds elicit activity in the fronto-temporoparietal (ventral) attention network in all participants but more strongly in ADHD patients. We aimed to identify the beneficial relationships underlying these novelty related activations and task performance.

## Materials and Methods

The local ethics committee of the university, faculty of medicine, approved the current study and confirmed its accordance with the ethical standards of the Declaration of Helsinki. All children and adolescents as well as their parents gave written assent/consent. The participants were reimbursed with 5€ - gift vouchers per hour for a local shopping center.

### Participants

Fifty-five boys aged between 11 and 16 years took part in the study. Of them, three patients and two typically developing participants (TD) had to be excluded: In both TD participants and one patient with ADHD we incidentally found brain abnormalities (pineal cysts). Two more patients with ADHD performed at chance level, which we interpreted as either lack of motivation or severe misunderstanding of the task. Thus, the final sample consisted of 25 ADHD patients and 25 typically developing children and adolescents (Table 1). All participants were either referred to us by the clinic of child and adolescent psychiatry or recruited via advertisement in a local newspaper. The diagnostic procedure included a semi-structured clinical interview, namely the German adaptation (Delmo et al., 2000) of the Revised Schedule for Affective Disorders and Schizophrenia for School-Age Children: Present and Lifetime Version (K-SADS-PL, Kaufmann et al., 1996) to assess clinical symptoms according to DSM-IV. The interview was conducted separately with every participant and his parent(s) by a trained psychologist. Accordance of all diagnoses with DSM-5 criteria was later ensured. Additionally, we assessed clinical symptoms *via* questionnaires by self-rating (YSR, Achenbach, 1991a) and parental judgement (CBCL, Achenbach, 1991b). Furthermore, intelligence (CFT 20-R, Weiss, 2008) and attentional capacity (d2-Test, Brickenkamp, 2002) were tested in all participants. Exclusion criteria were any current or previous neurological/psychiatric disorder other than oppositional defiant disorder or conduct disorder in the ADHD group, an IQ below 80, hearing impairments, evidence of substance abuse and medication intake other than stimulants in the patients.

**Table 1:** Description and group comparison of ADHD and typically developing (TD) participants.

	**ADHD**	**TD**	***t* (*p*)**
Number	25 males	25 males	
Age (years)	13.28	13.6	0.7 (.49)
IQ (CFT 20R)	96.58	106.7	3.59 (.0007)
Attentional performance (d2; T)	59.08	79,16	2.72 (.009)
Attentional problems, self rating (YSR; T)	55.25	40.32	2.31 (.02)
Attentional problems, parental rating (CBCL; T)	66.9	52.68	8.2 (< .0001)
Oppositional defiant disorder	5		
Stimulant medication: current (lifetime)	12 (16)		

Twelve patients were currently on stimulant medication (11 methylphenidate / 1 dexamfetamine) and discontinued the intake at least 24 h before and during the experiment. Four ADHD patients previously took stimulants (discontinued on average for 2 years) and nine were medication naïve.

Twenty participants were diagnosed with the combined subtype of ADHD, three with the predominantly inattentive subtype and two with the predominantly hyperactive-impulsive subtype. Furthermore, six participants in this group met diagnostic criteria for a comorbid oppositional defiant disorder (ODD). The comparison group of TD children paralleled the ADHD group in terms of age but displayed significantly higher IQ values and scores of attentional abilities (Table 1).

### Task and Procedure

**Figure 1:**
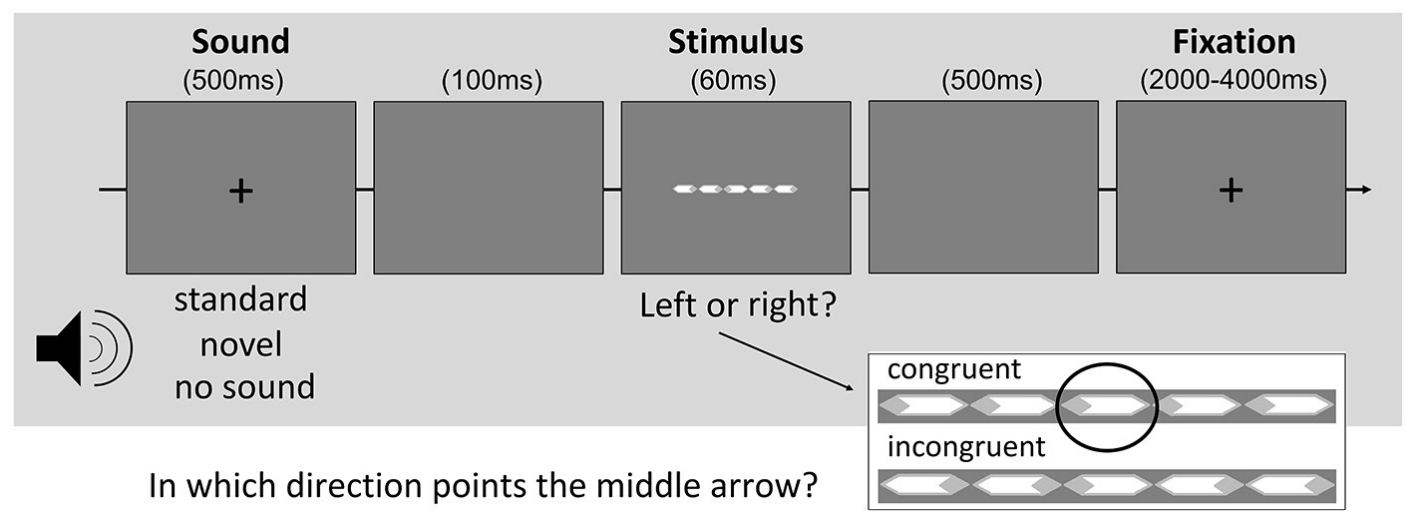
Illustration of the cued flanker task.

The behavioral task consisted of a modified version of the Eriksen flanker task (Eriksen and Eriksen, 1974) cued by auditory stimuli. In the flanker task, five white arrows surrounded by light-gray rhombi were presented on gray background (Figure 1). Participants had to indicate in which direction the central arrow pointed. The flanking arrows on each side could either be in alignment with the target (congruent condition) or point into the opposite direction (incongruent condition). Both conditions appeared with equal frequency. In 2/3 of all trials the visual task was preceded by either a repeatedly presented “standard” sound (1/3) or a unique “novel” sound (1/3). When no sound appeared, the fixation cross vanished for 500 ms in the respective time slot to give a visual indication of the upcoming task.

All auditory stimuli were environmental sounds selected from a German commercial CD (“1.111 Geräusche”, Döbeler Cooperations, Hamburg, Germany) cut to be of equal duration of 500 ms and edited to match in volume (60 dB) using the software Audacity (www.audacity.sourceforge.net). We further assessed the individual measures of spectral content (spectral central of gravity and pitch), spectral structure (harmonics-to-noise ratio) and temporal variability (standard deviation of frequency and amplitude) of each sound stimulus via Praat software (www.praat.org) (cf. Leaver and Rauschecker, 2010). Based on these parameters, those sounds were identified which achieved values within the second and third quartile of the respective distribution. This procedure resulted in a selection of six sounds representing stimuli of medium attentional salience compared to all other sounds. For each participant, one of them was randomly selected and used as repeatedly presented standard sound. The remaining 140 sounds served as uniquely presented novel sounds.

Each trial started with the sound presentation or disappearance of the fixation cross. Following these cues by 100 ms, the arrows appeared for 60 ms. Subsequently, a blank screen was presented for 500 ms before the fixation cross re-emerged. The inter-trial interval varied between 2 and 4 s (Figure 1). Participants were allowed to respond to the target for up to a maximum of 2 s. Later responses were treated as omission errors.

The experimental session started with a training block of the task (24 trials) outside the scanner. During training, novel sounds were not included, but half of the trials were preceded by the individual standard sound. Participants had the opportunity to repeat the training, if necessary and to ask questions. In the scanner, a comfortable volume level was adjusted under scanner noise individually for each participant. Then, participants performed three experimental task blocks with 120 trials each (40 trials per sound condition). Visual and auditory stimulation as well as recording of responses took place *via* Presentation software (www.neurobs.com).

### fMRI Image Acquisition and Processing

FMRI scanning took place on a 3T Philips Achieva dStream using a 32-channel head coil. A high resolution individual T1-weighted structural image was first collected in 192 sagittal slices with a voxel size of 1 mm3. Then, 290 whole-brain functional images were measured for each experimental run. An echo planar imaging (EPI) sequence parallel to the anterior-posterior commissure in axial planes was utilized. The sequence acquired 35 slices in ascending order with a spatial resolution of 3 mm^3^ (TR = 2 s, TE = 30 ms, FOV = 240 × 240, flip angle = 90).

Preprocessing and statistical analyses of the functional images was realized with the Statistical Parametric Mapping software (SPM12, Wellcome Trust Center for Neuroimaging, London) running on Matlab Version 2021b (the Mathworks Inc, MA). Preprocessing with SPM encompassed realignment to the first image in the respective run and co-registration of all images to the individual mean. Functional images were then spatially normalized to the anatomical T1 template provided by SPM (normalized voxel size of 2 mm3) and smoothed with a 6 mm FWHM istotropic Gaussian kernel. A highpass filter was applied at a cutoff of 1/128 Hz to remove low-frequency drifts.

### Behavioral Statistics

The behavioral measures of interest were changes in accuracy (percentage of correct trials), mean reaction time (RT) and the coefficient of reaction time variability (standard deviation divided by mean reaction time, RTV) for novel and standard sounds vs. the baseline without sound. First, we used linear mixed effects models to investigate the overall effects of the sound manipulation and ADHD on accuracy, RT and RTV. All models were computed in R statistics with the *lmer()* function of the lme4 package and included a random intercept for participants as well as age as covariate of no interest. The fixed main effects and interaction of the factors *Sound* (novel vs. standard vs. no) and *Group* (ADHD vs. TD) were modeled and tested using Wald tests (Type-II ANOVA tables) in the car package. If applicable, *post-hoc t*-tests were calculated. In a second step we directly tested for differences between standard and novel sounds minus baseline with the same model approach [fixed effects for *sound* (novel-no, standard-no) and *group*].

Furthermore, partial correlations controlling for age (ppcor package) were calculated to investigate the relationships between changes in task performance (accuracy, RT, RTV) and changes in neural signaling between novel and standard sounds in areas of interest as well as between these measures and IQ, d2-task performance and ADHD symptoms (parental and self-ratings of attentional problems), respectively.

### fMRI Statistics

Statistical analysis with SPM was based on the general linear model (GLM) approach. Individual GLMs included the three effects of interest (three sound conditions) and two effects of no interest (button press, false/missed responses) as well as 24 additional nuisance regressors based on the co-registration step during preprocessing to account for volume-to-volume head motion (three translation and three rotation, their squares, their derivatives, and squared derivatives). Moreover, the absolute difference between odd and even slices and slice variance were used to estimate within-volume motion for each TR. These regressors were included as further confounds in the GLM and were used to identify excessive motion (>4 × SD) in single volumes. These volumes were excluded using additional volume-specific regressors. Finally, the canonical hemodynamic response function time-locked to the onsets of the flanker task display was used to convolve the regressors. Then, individual contrast images were generated for the contrasts of interest to be used for second level group statistic.

Second level analysis of the fMRI data aimed on the one hand to investigate the neural basis of beneficial sound effects particularly in children and adolescents with ADHD. On the other hand, we wanted to disentangle the effects of alerting by standard sounds and alerting/orienting by novel sounds during task performance in general. Therefore, we first inspected group-wise activations associated with standard sounds as well as novel sounds compared to the baseline without sounds in each group. In the same manner, we determined the specific effect of novelty by contrasting novel with standard sounds per group (all one-sample *t*-tests). Then, we used two-sample *t*-tests to compare participants with and without ADHD regarding activity during standard > no sound baseline, novel > no sound baseline as well as novel > standard sound trials. An additional age regressor was included in all group contrasts to account for the wide developmental range. All contrasts were initially calculated with a threshold of *p* < 0.001 (uncorrected). The results were then corrected for multiple comparisons using a cluster-level false discovery rate (FDR) of *p* < 0.05.

To moreover examine possible relationships between neural activation and task behavior (RT, accuracy), we computed the contrasts of interest over all participants, extracted percentages of signal change from the peak voxel and calculated partial Pearson's correlations between these measures controlling for age.

## Results

### Sound Effects on Performance

**Figure 2:**
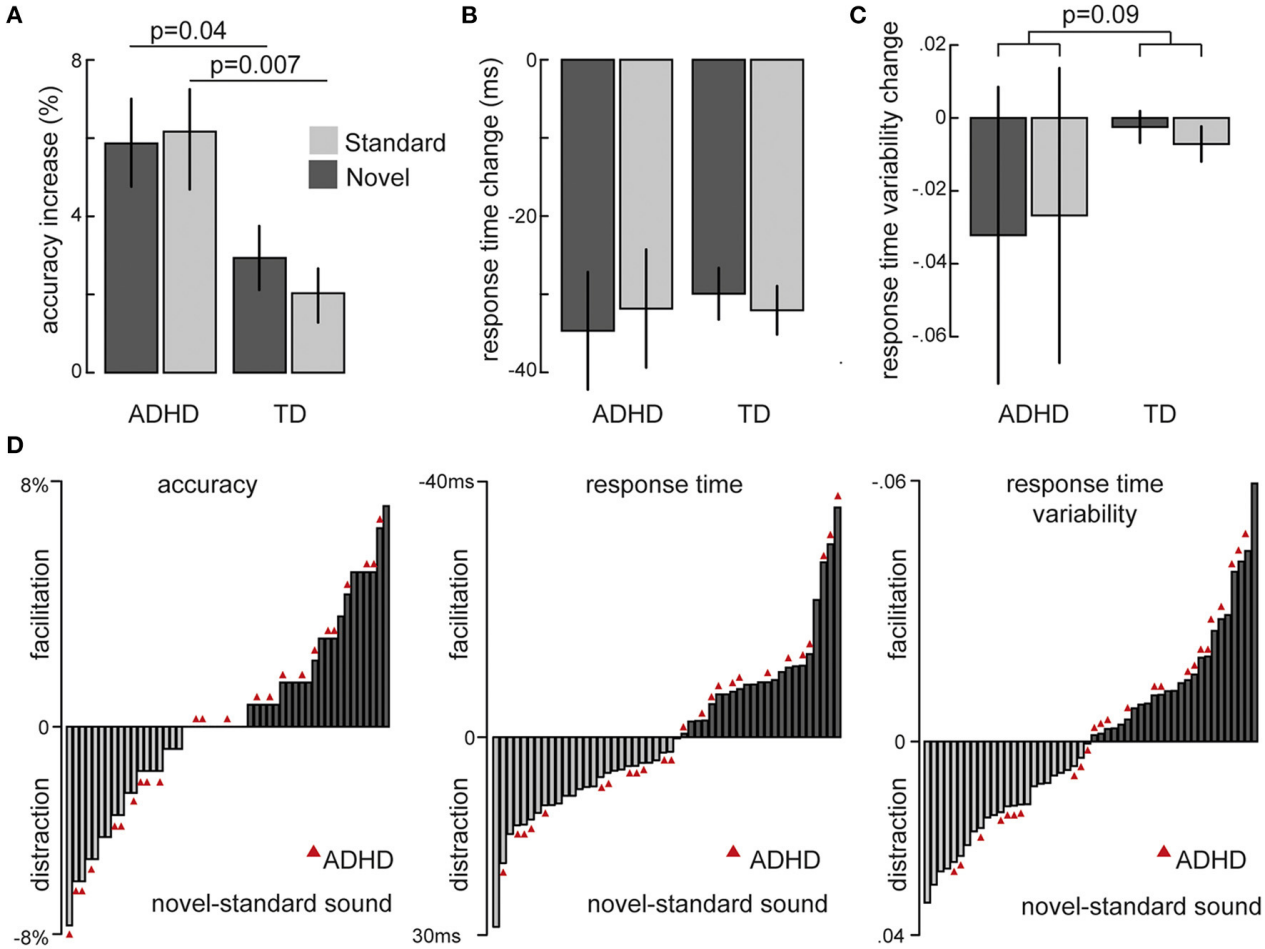
Overview of sound-induced changes in behavior from the baseline without sound separately for children and adolescents with ADHD and typically developing peers (TD): **(A)** Overall accuracy **(B)** Mean reaction times **(C)** Coefficient of reaction time variability (SD/Mean). **(D)** Individual differences for changes by novel minus standard sounds in all measures of interest. ADHD participants are highlighted by red triangles.

An overview over the changes in task performance from baseline by novel and standard sounds in children and adolescents with and without ADHD is depicted in Figure 2. The linear mixed effects model on accuracy over all three sound conditions showed that patients with ADHD made overall more errors that their TD peers [main group: $\chi_{\left(1\right)}^{2}$ = 11.01, *p* = 0.0009] and that sounds had a significant influence on accurate responding [main sound: $\chi_{\left(2\right)}^{2}$ = 56.64, *p* = 5.02e-13]. The repeatedly presented standard [t_(49)_ = 5.22, *p*= 3.5e-06] as well as the novel sound [t_(49)_ = 6.07, *p*= 1.8e-07] improved accuracy compared to the baseline without sound. Moreover, the effect of both sound conditions was elevated in the ADHD compared to the TD group [interaction: $\chi_{\left(1\right)}^{2}$ = 10.594, *p* = 0.005].

Average response times were accelerated by both sound conditions compared to no sound [main sound: $\chi_{\left(2\right)}^{2}$ = 111.5, *p* < 2.2e-16] and participants with ADHD responded overall more slowly than TD children and adolescents [main group: $\chi_{\left(1\right)}^{2}$ = 12.62, *p* = 0.00038]. The coefficient of response time variance (RT) was not influenced by group or sound as defined in the model (all *p* > 0.13). When pooled together, we however did see a significant decrease in RTV for sounds vs. no sound baseline [t_(69)_ = 2.94, *p* = 0.005].

To further explore the individual changes of novel and standard sounds and control for differences in the baseline, we used the respective differences to the no sound baseline for a direct comparison between novel and standard sounds. These models confirmed the overall greater increase in accuracy of both sounds on participants with ADHD (on average 6% improvement) compared to their typically developing peers (on average 2.48% improvement) [$\chi_{\left(1\right)}^{2}$ = 6.25, *p* = 0.012]. But they also revealed no differences between novel and standard sounds [$\chi_{\left(1\right)}^{2}$ = 1.45, *p* = 0.228] nor did we see differential effects per group [$\chi_{\left(1\right)}^{2}$ = 1.45, *p* = 0.228].

Average differences in response time were not influenced by either sound nor group (all *p* > 0.18) but we found a trend of an interaction between *sounds* and *group* on RTV [$\chi_{\left(1\right)}^{2}$ = 2.87, *p* = 0.09]. This trend resulted from a tendency of RTV to be reduced more by novel compared to standard sounds in the patients with ADHD whereas TD peers displayed the opposite trend of an increased benefit from standard sounds (Figure 2C).

Figure 2D depicts whether individual performance (accuracy, RT and RTV) was facilitated by novel compared to standard sounds and illustrates the considerable variance in the sample, both for participants with and without ADHD. We then tried to explain part of this variance with age, IQ, and ADHD symptomatology, respectively, but none of the measures showed any correlation with task performance (all *p* > 0.2). None of these results changed significantly when only ADHD patients with combined presentation were included (*N* = 20).

### Sound Effects on Neural Activity

**Figure 3:**
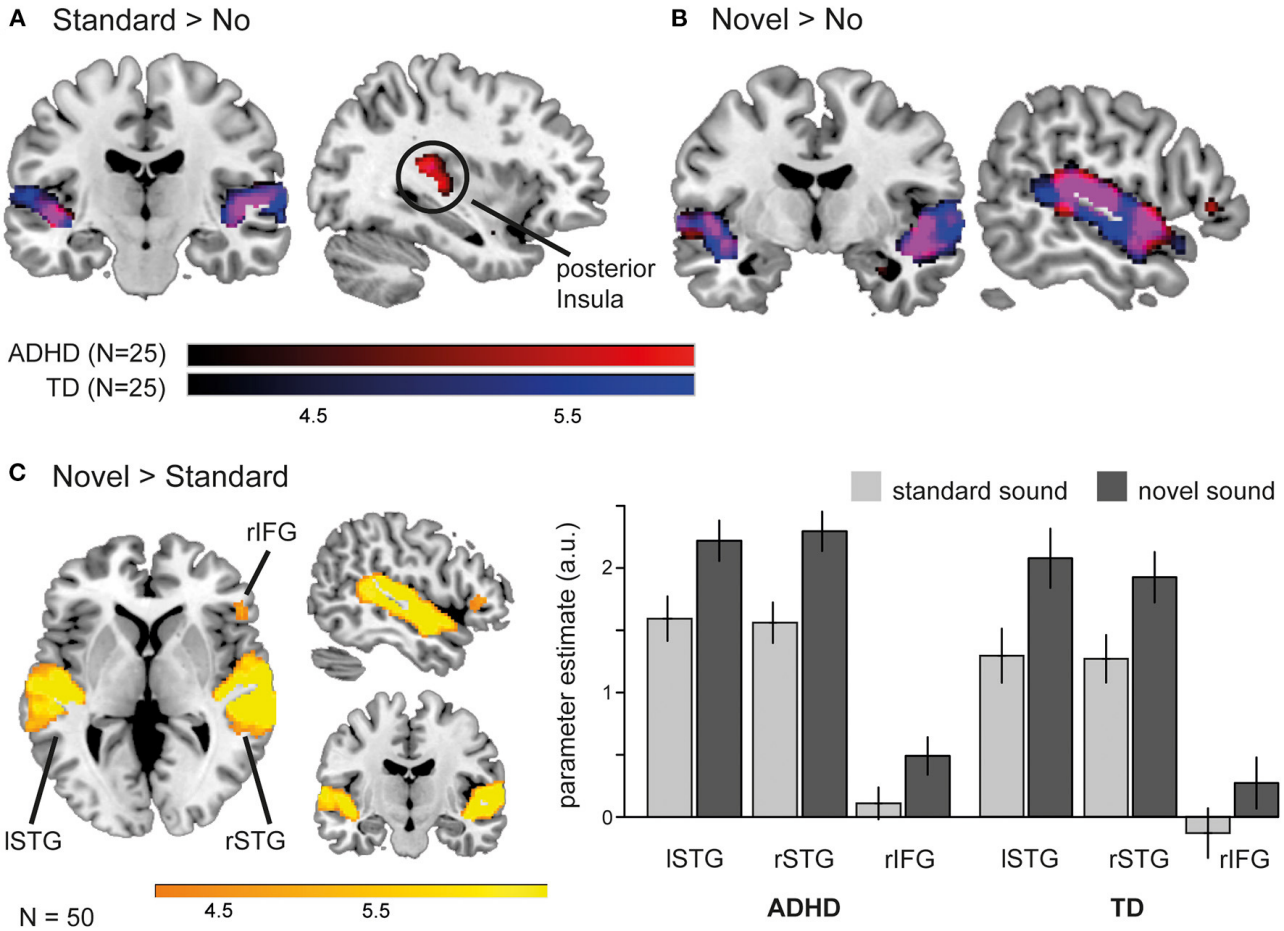
The influence of task preceding sounds on neural activation pattern during task execution. **(A)** Standard sounds modulated brain activity in ADHD patients (red) and TD peers (blue) in the bilateral superior temporal gyri (STG), but involved the posterior insula only in the patient group. **(B)** Novel sounds activated an overlapping (purple) network in children with (red) and without (blue) ADHD when compared to a baseline without sound (*p* < 0.001). **(C)** Direct comparison between novel and standard sounds revealed that novelty causes a significant increase in neural activity in the bilateral STG and right inferior frontal gyrus (rIFG) in all participants.

The effects of novel and standard sounds on neural activity during task execution were first examined *via t*-tests in each group (one-sample) and then compared between groups (independent sample) (Table 2). For both groups we found enhanced activity in the bilateral superior temporal gyri for standard sound trials compared to trials without a sound (Figure 3A). A similar pattern appeared when novel sounds preceded the task screen: participants with and without ADHD displayed increased activity compared to the baseline without sound in a large cluster encompassing the bilateral superior and middle temporal gyri extending into the anterior temporo-parietal junction (TPJ) and in patients with ADHD in the right inferior frontal gyrus (Figure 3B). The final comparison between novel and standard sounds revealed similar activation pattern in the bilateral STG and right IFG in both groups. Here the rIFG cluster survived FDR correction only in the TD group.

**Table 2:** Activation peaks (FDR corrected, *p* < 0.05) of group-wise and between group comparisons for sound related changes in brain activity during task performance in children and adolescents with ADHD and without (TD).

	**ADHD**					**TD**				
	***k***	**MNI (mm)**			***Z***	***k***	**MNI (mm)**			***Z***
**Novel** **>** **No Sound**										
R superior temporal gyrus	3,669	50	−26	8	6.82	4,265	56	−18	8	7.17
L superior temporal gyrus	3,337	−46	−18	−2	6.62	3,531	−50	−24	6	6.03
R inferior frontal gyrus	33	56	28	2	4.26					
**Novel** **>** **Standard Sound**										
R superior temporal gyrus	2,610	62	−36	6	6.51	4,226	60	−26	12	7.00
L superior temporal gyrus	1,963	−60	−32	10	5.57	3,175	−44	−30	10	7.06
R inferior frontal gyrus						92	52	18	22	4.15
**Standard** **>** **No Sound**										
R superior temporal gyrus	2,003	56	−22	8	6.25	2,271	62	−18	6	6.03
L superior temporal gyrus	2,062	−44	−20	0	5.94	2,051	−44	−30	6	6.10
R Hippocampus	61	32	−8	−22	4.86					
	*ADHD > TD*									
L post. insula/Putamen	182	−30	−10	6	4.11					

In line with these group wise contrasts, group comparisons revealed no difference between the groups in brain activation following novel sounds (neither when compared to the baseline without sound nor to standard sounds). However, the comparison of activity pattern evoked by standard sounds during task execution (standard > no) revealed enhanced activation for ADHD patients compared the TD peers in the left posterior insula extending to the putamen (depicted in the group clusters in Figure 3A).

As we could not detect novelty related differences in neural activity between children and adolescents with and without ADHD, we collapsed over all participants (*N* = 50) to identify a common network of novelty-related brain activation during task execution (Figure 3C). This *t*-test revealed activation in the left (−60, −32, 10; *k* = 3,905 T = 11.87) and right STG (64, −26, 10; *k* = 4,580, T = 14.26) as well as rIFG (52, 30, 4; *k* = 517, T = 5.30) for the whole sample (*p* < 0.05, FDR corrected).

**Figure 4:**
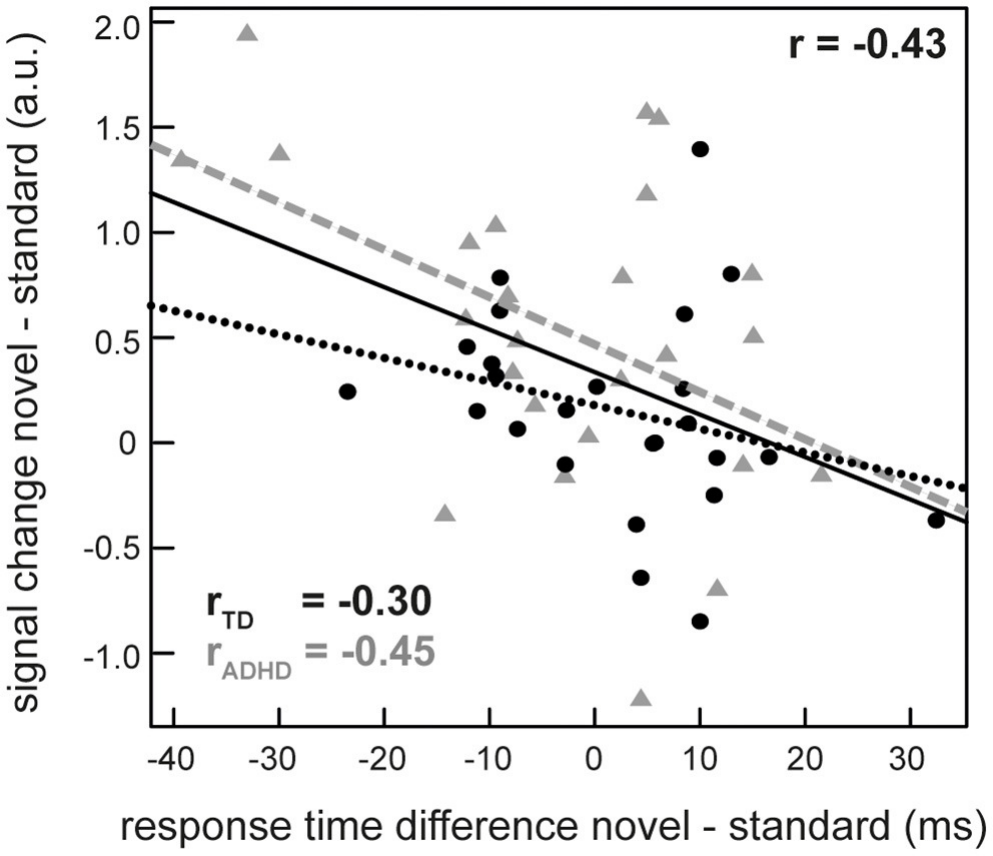
Correlation between the activity difference between novel and repeatedly presented sounds in the right inferior frontal gyrus (rIFG) and the difference in reaction times in these conditions in the whole sample (solid line) as well as separately for ADHD patients (gray triangles, dashed line) and typically developing (TD) participants (dots, dotted line).

Next, we extracted individual percentages of signal change from the peak voxel of these areas (novel > standard) and used these parameters to identify correlations between brain activity and task measures of interest (changes in accuracy, mean RT and RTV for novel—standard sounds). We found a significant negative correlation between neural activity in the rIFG and the difference in mean RT across all participants (*r* = −0.42, *p* = 0.002). Exploratory inspection of this relationship between rIFG activity, group and RT revealed that the correlation was based upon the participants with ADHD. When considered alone, the patient group showed a correlation of *r* = −0.45 (*p* = 0.02). TD peers, on the other hand, displayed a weaker relationship that failed statistical significance (*r* = −0.30, *p* = 0.15) (Figure 4). No association between neural activity and RTV, accuracy and ADHD symptoms or between activity in the bilateral STGs and behavior/symptoms could be detected.

## Discussion

The current study aimed to identify activity in neural attention networks that underlie the beneficial effects of task preceding sounds on subsequent performance in children and adolescents with and without ADHD. We were particularly interested in the effects of unique (novel) sounds compared to a repeatedly presented standard as both types of sounds should function as alerting cues, but only novel sounds were supposed to elicit an orienting response.

We found that novel sounds caused activity in large bilateral clusters spanning the STGs extending to anterior TPJs as well as the right IFG in all participants, without detectable alterations in patients with ADHD. This network is in line with former descriptions of the ventral attention (orienting) network (VAN) (Corbetta and Shulman, 2002; Corbetta et al., 2008; Petersen and Posner, 2012) but also covers the bilateral auditory cortex. In contrast to most previous reports, activation in the fronto-temporoparietal VAN was not paralleled by disruption of task performance in either group. Instead, novel sounds had a beneficial effect on reaction times and accuracy compared to the baseline without sound. Moreover, the difference between rIFG neural activity for novel minus standard sounds was correlated with the benefit in speed for novel vs. standard sound trials during the flanker task. This relationship indicates a direct facilitating effect of brain activity on subsequent task behavior caused by novelty.

Importantly, although the increase in accuracy for novel sounds in patients with ADHD exceeded the effect of novelty in TD peers, the ADHD group did not display a stronger neural orienting response than the comparison group. This neural activation overlap is in contrast to reports of enhanced sensitivity for novelty in ADHD patients in oddball tasks (Gumenyuk et al., 2005; Tegelbeckers et al., 2015). In these tasks, novelty processing is part of the task and thus influenced by potential motor reactions and target identification. Ongoing executive processes such as response selection, execution, and/or inhibition make it therefore difficult to disentangle attentional orienting effects of novelty from task related processes. Top-down attentional processes should be significantly more involved in studies using oddball designs compared to the current setup and could be the source of ADHD related impairments (Tegelbeckers et al., 2015) rather than enhanced bottom-up reactions of the fronto-temporo-parietal VAN. Moreover, the occurrence probability of a novel stimulus is significantly reduced in oddball tasks compared to the current study in which we presented novel sounds with the same probability as standard sounds. As the influence of novel stimuli possibly depends on the degree of their deviation from context (Schomaker and Meeter, 2015), group differences might become more prominent with lower frequency of novel events (Tegelbeckers et al., 2015). It is therefore likely that the context in which novel sounds occur (or orienting in general is provoked) plays an important role for alterations in ADHD. Moreover, our current finding of intact neural orienting following novel sounds in ADHD is in line with other previous reports of efficient utilization of orienting cues in the visuo-spatial domain in patients with ADHD (e.g., Huang-Pollock and Nigg, 2003; Oberlin et al., 2005; Booth et al., 2007; Mullane et al., 2011). Evidence accumulates that these patients are not impaired in filtering irrelevant sounds when engaged in a task (Friedman-Hill, 2010) and are even able to use these sounds to improve performance (van Mourik et al., 2007; Tegelbeckers et al., 2016).

We thus combined the groups for a total of 50 participants to gain a new perspective on the potential general neural mechanisms underlying these task improvements: When we and others showed behavioral facilitation by novel sounds before (van Mourik et al., 2007; Tegelbeckers et al., 2016), we argued that the alerting benefits associated with novelty might cause these improvements. Benefits in terms of lower reaction times or higher accuracy that are associated with activity in the fronto-temporopartietal VAN seem to contradict the assumed model of “*orienting costs*” and “*alerting benefits*” (SanMiguel et al., 2010). Still the novelty related activations in the bilateral STG and rIFG overlap largely with the previously described “orienting network” (Corbetta et al., 2008; Petersen and Posner, 2012).

Here, we propose that at least three explanations can account for beneficial effects of orienting network activity during task performance. First, activation associated with attentional orienting might carry an internal alerting effect in itself that is independent of the alerting network but might also be modulated by noradrenergic pathways. Corbetta et al. (2008) suggested a functional connection between noradrenergic locus coeruleus neurons and the fronto-temporoparietal VAN because both systems show similar activation pattern during deviance detection. Alternatively, the distracting effect of attentional orienting might be time dependent. Our delay of 600 ms between sound onset and target appearance might have enabled the redirection of attention without any behavioral cost. An orienting response concurrent with target presentation might have a different (more distracting) effect (Berger and Cassuto, 2014). Finally, the role of the fronto-temporoparietal VAN might as well not be limited to attentional orienting. This presumption is supported by investigations showing that VAN activity following sensory stimuli is driven by task-relevance rather than stimulus salience (Corbetta et al., 2008). As these findings contradict pure bottom-up transfer, it has been speculated that these signals indicate transitions between two tasks or behaviors (for review see Corbetta et al., 2008 or Vossel et al., 2014). Thus, as novel sounds in the current experiment serve as temporal cues announcing the task, fronto-temporoparietal VAN activity during the flanker task might reflect contextual updating of the switch between auditory and visual processing. The effects of novel sounds might then indeed rely on VAN activity characterized as “orienting response” in so far as the current task set is re-installed and behavioral execution is facilitated.

Interestingly, within the network the rIFG seems to play a crucial role of directly controlling behavior as rIFG activity was correlated with the benefit in reaction times. This fits the proposed role of the rIFG as a hub for executive control, particularly for successful attentional control and inhibition (Hampshire et al., 2010). It seems likely that the increase in attentional control *via* activation of the rIFG which is caused by orienting toward novelty is responsible for the benefits in task performance we observe in this setup. Moreover, structural (Sowell et al., 2003; Durston et al., 2004) and functional (Aron and Poldrack, 2005; Rubia et al., 2011) alterations of the rIFG are commonly reported in patients with ADHD, indicating a particular role of this region in the attentional problems associated with the disorder. Our findings of a modulation of task performance by an increase in rIFG activity further supports the choice of this area as a target for non-invasive brain stimulation (Breitling et al., 2016).

Finally, in addition to the novelty related findings, we also investigated the alerting effects of the frequently presented standard sound. Standard sounds improved accuracy and accelerated the responses to a similar extent as novel sounds. The ADHD group seemed to particularly benefit in accuracy also by the standard which illustrates once more that the disorder is characterized by an underlying alerting deficit (Sergeant, 2000). The lower general level of alertness probably enabled ADHD patients to improve to a greater extent than TD peers in trials which contained a warning stimulus. Moreover, the greater behavioral alerting benefits in the ADHD compared to TD group were paralleled by enhanced neural activity for standard sound trials vs. the baseline without sound in the left insula extending to putamen. As the insula is known to be involved in the integration of salient information and coordination of attentional resources (Menon and Uddin, 2010), insular dysfunction during cognitive tasks has been associated with ADHD before (Cortese et al., 2012). Here, we showed that repeatedly presented sound cues could increase insular activation and potentially normalize behavioral performance. Moreover, both groups showed activity pattern in the bilateral STG and auditory cortices for trials with preceding standard cue which provides further evidence for the idea that “alerting network” activity depends on modality specific brain areas that rely on the respective primary sensory cortices and processing pathways of the cue modality (De Santis et al., 2007; Langner et al., 2012; Spagna et al., 2015).

Taken together, the current experiment investigated brain responses associated with the beneficial effects of task-preceding standard and novel sounds in children and adolescents with ADHD and a typically developing comparison group. Although carefully designed, the study in its current form still faces some limitations. First, it is possible that we failed to find novelty related differences between ADHD and TD participants due to the sample size (type II error) or specific characteristics of the ADHD sample. We only included males and about half of our ADHD sample had used stimulant medication over the lifespan. Twelve patients currently took medication and although they discontinued the intake for at least 24 h, it cannot be ruled out that responsivity to the task preceding tones is altered in comparison to medication naïve participants.

Overall, we showed that sounds acting as warning cues can improve performance in all participants but particularly in ADHD patients. Moreover, and potentially caused by an underlying disorder-specific alertness deficit (Sergeant, 2005), ADHD patients showed increased brain activations during task execution when familiar sounds were presented. We found no evidence for altered processing of novel task preceding sounds, indicating that in this setup ADHD patients showed no higher distractibility than TD peers. Instead, the neural orienting response following novel sounds was unimpaired in the patient group and rIFG activity directly improved task performance.

Thus, our results are encouraging in terms of the identification of optimized learning and working conditions for children and adolescents with ADHD. Their increased susceptibility to beneficial effects of alerting sounds might be used in potential treatments, either by e.g. incorporating external stimulation into homework situations or even by targeting the rIFG and/or left insula with transcranial electrical stimulation. Moreover, the individual reactivity to novelty could potentially be used to identify subtypes of attention profiles in patients with ADHD.

## Data Availability Statement

The datasets presented in this article are not readily available because permission to share individual (f)MRI data from the underage study participants was not obtained for ethical reasons. Behavioral data, group fMRI data and code will be made available upon reasonable request from the corresponding author. Requests to access the datasets should be directed to jana.tegelbeckers@ovgu.de.

## Ethics Statement

The study was reviewed and approved by the Local Ethics Committee of the Otto von Guericke University, Faculty of Medicine. Participants and their legal guardian received a detailed oral and written information about the study procedure. Written informed consent to participate in this study was provided by the participants' legal guardian/next of kin. Participants were also asked to provide written assent.

## Author Contributions

JT, AB, BB, H-HF, and KK designed the study. JT and CB-Z performed the experiment. JT and KK analyzed the data and wrote the manuscript. AB, CB-Z, BB, and H-HF edited the manuscript. All authors contributed to the article and approved the submitted version.

## Funding

This work was supported by the Deutsche Forschungsgemeinschaft (Sonderforschungsbereich 779, TP A03).

## Conflict of Interest

The authors declare that the research was conducted in the absence of any commercial or financial relationships that could be construed as a potential conflict of interest.

## Publisher's Note

All claims expressed in this article are solely those of the authors and do not necessarily represent those of their affiliated organizations, or those of the publisher, the editors and the reviewers. Any product that may be evaluated in this article, or claim that may be made by its manufacturer, is not guaranteed or endorsed by the publisher.
